# Transcriptomics-Based Toxicological Study of Nickel on *Caenorhabditis elegans*

**DOI:** 10.3390/toxics13110930

**Published:** 2025-10-30

**Authors:** Yutao He, Yunfei Long, Jingwen Wang, Qinfen Li, Beibei Liu, Dandan Li, Shunqing Xu

**Affiliations:** 1 School of Life and Health Sciences, Hainan University, Haikou 570228, China; 222208600081@hainanu.edu.cn (Y.H.); 13657475976@163.com (Y.L.); 20233000827@hainanu.edu.cn (J.W.); 2 Environmental and Plant Protection Institute, Chinese Academy of Tropical Agricultural Sciences, Haikou 571101, China; qinfenli2005@163.com (Q.L.); liubeibei1110@126.com (B.L.); 3 School of Environmental Science and Technology, Hainan University, Haikou 570228, China

**Keywords:** Ni, *Caenorhabditis elegans*, developmental arrest, lipofuscin, transcriptomics

## Abstract

Nickel (Ni), a heavy metal with extensive industrial applications, poses significant ecological impacts and health risks due to its persistence and bioaccumulation. Although toxicological data in mammals and plants are well established, its effects on invertebrate models remain insufficiently explored, especially at environmentally relevant concentrations. This study systematically evaluated the toxicity of Ni^2+^ on *Caenorhabditis elegans*, integrating phenotypic assays with transcriptomic profiling to assess impacts on growth, reproduction, neuromuscular function, lifespan, and aging. Ni exposure induced dose-dependent developmental delays. After exposure to 80 μg/L Ni^2+^ for 72 h, the proportion of L1-stage nematodes increased 3.8-fold compared to the control group. Similarly, exposure to 80 µg/L Ni^2+^ reduced the reproductive capacity of nematodes to 88.5% of that in the control group. Transcriptomic analysis identified 2235 differentially expressed genes (DEGs) after 8 μg/L of Ni^2+^ exposure, while the worms exposed to 0.8 μg/L of Ni^2+^ exhibited a total of 249 DEGs. GO (Gene Ontology) and KEGG (Kyoto Encyclopedia of Genes and Genomes) analyses highlighted collagen metabolism defects, fatty acid-related metabolism, amino acid-related biosynthesis disruption, and lysosomal dysfunction, correlating with cuticle integrity loss, energy metabolism abnormality, and feeding behavior change, and indirectly lead to delayed growth development and lipofuscin accumulation. The latter is usually regarded as a reliable indicator of aging, suggesting that exposure to Ni poses a risk of accelerating aging in nematodes. This study provides critical insights into the ecological risks of Ni pollution.

## 1. Introduction

Nickel (Ni) is a hard and ductile transition metal, exhibiting superior physical and chemical properties [1]. Ni serves as a core component in stainless steel and alloy steel and is widely utilized in the electroplating, aerospace, and military industries [2]. Over the past 30 years, global Ni production has witnessed a 370% increase, reaching 3300 kilotons (kt) in 2023 [3,4]. Furthermore, Ni possesses the characteristics of high conductivity and high energy density. It has been used as the positive electrode material for lithium-ion batteries and is widely applied in the power generation sector of clean energy [1]. It is expected that by 2030, the share of the battery industry in global nickel consumption will increase from 7% in 2021 to 37%, ranking second only to its share in the steel industry [5]. However, the rapid development of the Ni industry has greatly accelerated the dispersion of Ni into the air, water, and soil environments. In the atmosphere of urban areas, the average concentration of Ni varies from 3 to 30 ng/m^3^ [6], while it varies from 70 to 770 ng/m^3^ in heavily industrialized areas [7]. Ni content in contaminated soils ranges from 3 to 1000 mg/kg [8,9]. Moreover, the Ni content in the surface soils exposed to industrial wastewater and sewage is usually higher than that in the subsurface soil. The level of Ni in contaminated aquatic resources is estimated to be 0.2 mg/L, which is roughly 20 to 40 times higher than that in unpolluted water sources [8,10,11]. Furthermore, the level of Ni in sediments can reach 10–300 mg/kg [12], which poses a long-term exposure risk to benthic organisms, including nematodes. Ni has four oxidation states (+1, +2, +3, and +4), with the +2 oxidation state being the most common in biological systems. Ni (II) is readily available and more toxic in cationic form than its complexes [8]. The physicochemical properties of Ni^2+^ and its interaction with biomolecules in the culture medium determine its bioavailability and toxicity. Generally, the order of human absorption of nickel compounds from highest to lowest is as follows: nickel carbonyl (Ni(CO)_4_), water-soluble nickel compounds, insoluble metallic nickel, and its compounds (e.g., Ni(OH)_2_ and NiO) [13]. Nickel carbonyl compounds are highly toxic. They can be rapidly absorbed by the body upon inhalation by both humans and animals, leading to acute poisoning [14]. Ni ions (Ni^2+^) have high bioavailability and can penetrate cell membranes to induce oxidative stress and mitochondrial dysfunction. Ni nanoparticles (5–100 nm) have both physical damage and chemical toxicity (continuous release of Ni^2+^ in acidic environments), with strong penetration and easy accumulation, resulting in a dual toxicity mechanism [15].

Previous studies have shown that Ni^2+^ exposure through diet, occupation, and everyday items can lead to human health issues, including allergies and cancer [8]. Ni contamination has become a concerning problem, particularly in developing countries, due to its non-degradability in the environment. According to the national soil pollution survey report of China in 2014, Ni pollution accounted for 4.8% of all soil pollution incidents, ranking among the top in terms of inorganic pollutants [16]. The traditional view holds that Ni in low concentrations has no adverse effects on human health, but exceeding concentrations of Ni could cause health risks in target consumers. When the Ni content in soil and water exceeds 35 mg/kg and 0.02 mg/L, respectively, it will cause toxicity to all living organisms [9]. Hence, Ni concentrations in various environmental compartments are regulated to mitigate ecological and human health risks. In drinking water, the World Health Organization (WHO) recommends a guideline value of 0.07 mg/L for Ni [17]. The WHO has recommended safe limits for Ni content in wastewater and agricultural soil, which are 0.02 ppm and 0.05 ppm, respectively [18]. However, whether low-concentration Ni exposure is truly safe still requires evidence to prove.

The toxicity issues of Ni (II) in microorganisms, plants, animals, and humans have attracted widespread attention [8,15,19]. The toxicity of Ni^2+^ to microorganisms is associated with oxidative stress, membrane damage, cell cycle dysregulation, metabolic disorders, and the displacement of essential metal ions that are required to maintain the activity of metalloenzymes [19]. For example, Ni^2+^ competitively inhibits ABH2, which is a nucleolus-enriched DNA alkylation repair enzyme in cells, by occupying the iron-binding site [20]. It has been found that long-term Ni exposure can alter the diversity of microbial communities in agricultural soils, and soil microbial biomass carbon (the total carbon stored in soil in form of microbes, a key measure of soil biological health and ecosystem function) consistently decreased along Ni gradients [21]. Although Ni is a necessary nutrient for the normal growth and development of plants, excessive exposure to Ni can cause physiological changes in plants by altering enzyme activity, triggering oxidative stress, and disrupting photosynthesis, ultimately affecting their growth and yield [22]. Furthermore, plants can absorb and transport Ni^2+^, which increases the risk of humans being exposed to Ni^2+^ through the food chain. Ni mainly enters the human body through skin contact/absorption, ingestion, and inhalation [23]. And excessive exposure to nickel has been proven to cause allergic reactions, cardiovascular diseases, pulmonary fibrosis, lung cancer, kidney diseases, and blood system disorders [8]. The U.S. Environmental Protection Agency has classified nickel sulfide dust generated by nickel refineries as Group A human carcinogens, while Ni metal and Ni alloys are classified as Category 2B (possibly carcinogenic to humans) [13]. For animals, Ni poisoning occurs through various mechanisms, such as impaired ion regulation function, respiratory inhibition, and the promotion of oxidative stress. Nickel-induced cell apoptosis or carcinogenesis is associated with the production of reactive oxygen species (ROS), lipid peroxidation, protein carbonylation, and DNA double-strand breaks. Previous studies have shown that exposure of a Chinese hamster cell line (G12 strain) to Ni_3_S_2_ at a concentration of 0.3 μg/cm^2^ for 24 h alters DNA methylation levels, ultimately leading to carcinogenesis [24,25,26]. Furthermore, Ni interferes with histone-related modification, altering chromatin structure and gene expression [27]. High doses of NiCl_2_·6H_2_O (administered via gavage at 30 mg Ni/kg body weight for 14 days) induce a decrease in the activity of mitochondrial respiratory chain complexes I-IV and ATP content in the mouse liver, and NiCl_2_ causes mitochondrial damage, characterized by increased mitochondrial ROS (mt-ROS) production and depolarization of the mitochondrial membrane potential (MMP) [28]. Broiler chickens exposed to nickel chloride at doses exceeding 300 mg/kg for 42 days exhibited oxidative stress in the cecal tonsils, resulting in decreased protein expression, mRNA expression, and protein content of *Bcl-2* while simultaneously increasing the protein expression, mRNA expression, and protein content of both *Bax* and *caspase-3* [29]. Ni refining dust (with a Ni content of 94.88% and diameter of <5 mm in 99% of particles) exposed for 24 and 48 h (with concentrations of 0, 25, 50, and 100 µg/mL) could promote the expression of NF-κB in mouse embryonic fibroblast cells (NIH/3T3 cells) and induce the secretion of inflammatory-related factors (TNF-α, IL-1β, iNOS, and COX-2) in a dose- and time-dependent manner [30]. Maternal intake of Ni (NiCl_2_·6H_2_O, gavage at doses of 46.125, 92.25, and 184.5 mg Ni/kg body weight) poses a persistent threat to offspring, which exhibit susceptibility during different pregnancy and lactation periods—namely the preimplantation, organogenetic, and fetal stages; among these, the organogenetic period is the most critical developmental window [31].

*Caenorhabditis elegans* (*C. elegans*), an internationally recognized model organism, offers numerous advantages including a well-defined genetic background, a short life cycle (approximately 3 days), and suitability for high-throughput screening. These characteristics make it an ideal model for environmental toxicology research. Many studies have demonstrated that *C. elegans* can be used for studies on toxicity of metals, such as studying the toxicity of zinc (Zn), copper (Cu), and cadmium (Cd) under single-metal and mixture scenarios at different organizational levels [32]. There have been quite a few studies on the toxicity of Ni, but most studies focus on mammalian cells or plants, with limited attention to model organisms like *C. elegans*. It is particularly crucial that current invertebrate research mainly focuses on acute endpoints at doses far above environmental levels (such as ≥1 mg/L in aquatic exposure), seriously ignoring the exposure effects under actual environmental field concentrations. After the treatment of *C. elegans* at the first larval (L1) stage for 1 h, NiCl_2_ (2 mol/L) induces neurodevelopmental toxicity, including cholinergic, dopaminergic, and GABAergic degeneration and behavioral alterations, and upregulates expression of glutathione S-transferase 4 (gst-4) [33]. Wang et al. [34] found that in *C. elegans*, the damage caused by nickel sulfate (NiSO_4_) exposure (at 2.5, 75, and 200 μM) can be passed on from the parents to the offspring. Tang et al. [35] found that NiSO_4_ affected growth (5–50 μmol/L), brood size (1–500 μmol/L), feeding (1–200 μmol/L), and locomotion (1–200 μmol/L) in a wild-type N2 *C. elegans* strain. The toxic effects of different types of Ni salts on Ni against nematodes have also been evaluated. Nickel fluoride (NiF_2_), anhydrous Ni iodide (NiI_2_), and Ni chloride (NiCl_2_) have the highest toxicity to *C. elegans*, followed by Ni nitrate (Ni(NO_3_)_2_), Ni sulfamate (Ni(SO_3_NH_2_)_2_), Ni acetate tetrahydrate (Ni(OCOCH_3_)_2_), and Ni sulfate (NiSO_4_) [36]. In halide solutions, the bioavailability of free Ni ions is higher than that of other substances. Therefore, the authors suggest that in future nickel content tests for aquatic invertebrates, halogenated salts should be used [36,37]. Accordingly, we used *C. elegans* as the model organism to evaluate the toxicity induced by Ni at environmentally related concentrations (0.8–80 μg/L) systematically, combined with transcriptomic analysis to elucidate its toxic mechanisms. The results of this study will provide a valuable supplement to the research on the ecological and health risks associated with Ni.

## 2. Materials and Methods

### 2.1. Chemicals and Raw Materials

Analytical reagent (AR)-grade nickel chloride hexahydrate (NiCl_2_·6H_2_O, 98%), sodium hypochlorite, potassium dihydrogen phosphate, and disodium hydrogen phosphate were purchased from Xilong Scientific (Guangzhou, China). AR-grade sodium hydroxide was purchased from Guangzhou Panyu Lihua Factory (Guangzhou, China). PBS powder in sachets was obtained from Servicesbio (Wuhan, China) and further dissolved using Milli-Q water (18.2 MΩ·cm at 25 °C, prepared by Direct-Q5, Merck Millipore, Merck Ltd., Beijing, China). Stock solutions of Ni^2+^ at 1000 mg/L were also prepared in MilliQ-filtered tap water (18.2 MΩ·cm, 25 °C), and work concentrations (0.8, 8 and 80 μg/L) were diluted from the stock using *Escherichia coli* (*E. coli*) OP50 bacterial solutions. Then, 100 μL of working solutions with different concentrations were respectively applied to the nematode growth medium (NGM) plates, dried, and set aside for further use, and 100 μL of OP50 without Ni^2+^ was used as control. OP50 bacteria were cultured with Luria–Bertani (LB) liquid medium. NGM plates, OP50, and LB liquid medium were prepared as previously described [38,39].

### 2.2. Cultivation and Synchronization of C. elegans

The *C. elegans* strains were sourced from the Caenorhabditis Genetics Center (University of Minnesota, Minneapolis, MN, USA). All samples of wild-type *C. elegans* Bristol strain N2 were maintained at 20 °C on NGM plates seeded with OP50 bacteria. Nematode developmental stages were monitored under a stereomicroscope (Jiangnan JSZ6, Jiangnan Yongxin, Nanjing, China). The gravid adult worms were lysed using a bleaching solution, and eggs were collected to obtain synchronous nematodes. Specifically, the gravid adult nematodes were washed from the plates into 1.5 mL microcentrifuge tubes by using M9 minimal medium buffer (1.0 g/L NH_4_Cl, 0.5 g/L NaCl, 0.241 g/L MgSO_4_, 3 g/L KH_2_PO_4_, 6.78 g/L Na_2_HPO_4_, 0.011 g/L CaCl_2_, 4 g/L glucose) and washed by centrifugation (Multifuge X1R Pro, Thermo Scientific, Waltham, MA, USA). Pelleted nematodes were treated with bleach solution (0.45 M NaOH, 2% HClO) for ~3 min. After centrifugation (6000 rpm, 1 min), pellets were washed twice with M9 buffer. Eggs were transferred to fresh OP50 seeded on new NGM plates for subsequent use.

### 2.3. Developmental Assay for C. elegans

Synchronized eggs were inoculated onto control (OP50 only, without Ni^2+^) and experimental NGM plates containing Ni^2+^ (0.8, 8, or 80 μg/L). After being cultured at 20 °C for 72 h, the number of nematodes in each stage (L1–L4) was observed and recorded. *C. elegans* progresses through four larval stages (L1 to L4) after hatching before reaching adulthood. Generally, L1 larvae (newly hatched) are tiny with no visible reproductive organs, L2/L3 actively feed, and L4 shows a pale band near the tail (indicating sexual maturation) and nears adult size but remains immature. This developmental sequence parallels human growth from infancy (L1) to adolescence (L4). At 20 °C, the transition from L1 to adult takes ~50 h, and scientists track stages by observing physical traits (e.g., gonad structure) under a low-magnification microscope or by conducting fixed-cycle cultivation based on known time benchmarks (e.g., the period from the L1 stage to the L2 stage is about 12 h and from the L2 stage to the L3 stage is about 8 h) [40,41].

### 2.4. Reproductive Capacity Assay

Well-developed nematodes were subjected to synchronization treatment to obtain a population at a uniform developmental stage. Then, the harvested eggs were respectively added to the NGM plates (control group and Ni^2+^ exposure groups). After being cultured for 72 h, the nematodes at stage L4 were selected and transferred to new NGM plates. Fifteen plates were set up in each group, and one nematode was placed on each plate. The number of eggs laid by the nematodes every 8 h was recorded until the nematodes stopped laying eggs. Furthermore, the eggs and larvae were scalded to death with a sterile platinum wire to prevent them from growing and affecting the count.

### 2.5. Lifespan Assay

Synchronized nematodes were randomly cultured on control or Ni^2+^ containing NGM plates (5 plates in each group). After the nematodes developed to the L4 stage, 12 nematodes were selected from each plate and transferred to fresh culture medium with corresponding Ni^2+^ concentration and 5-Fluorouracil (5-FU). The number of survivors was recorded daily until all animals died.

### 2.6. Lipofuscin Accumulation Assay

Lipofuscin is a type of spontaneous fluorescent pigment granule accumulated during the process of cellular aging. It is commonly used in the study of aging, oxidative stress, and mechanisms of lifespan regulation [42]. For the lipofuscin accumulation assay, the synchronized nematode eggs were randomly divided into four groups and were respectively cultured at 20 °C for 72 h in NGM plates containing Ni^2+^ (0.8, 8, or 80 μg/L) and those without Ni^2+^ (control). After cultivation, the lipofuscin in nematodes was observed and imaged using a Nikon stereomicroscope (ECLIPSE Ni-U, Nikon Corporation, Tokyo, Japan) with excitation at 425 nm and emission at 535 nm. Fluorescence intensity was quantified using ImageJ2 software. At least 20 nematodes were analyzed in each group.

### 2.7. Food Preference Assay

NGM plates were divided into two regions: Regions A (OP50) and B (OP50 + 80 μg/L Ni^2+^). Synchronized L4-stage nematodes were placed at the plate center and nematode distributions were recorded after 2 h (*n* = 30).

### 2.8. Transcriptomic Analysis

Transcriptomes of *C. elegans* were prepared as previously described [39]. Specifically, synchronized *C. elegans* was cultured on NGM plates with different Ni^2+^ treatments until reaching Day 3. Then, the nematodes were collected and washed by centrifugation (Multifuge X1R Pro, Thermo Scientific) and quickly frozen in liquid nitrogen for transcriptome sequencing analysis. The specific sequencing process can be found in the Supplementary File S1 for further details. Raw sequencing data were processed using FastQC v0.12.1 for quality control, aligned with the *C. elegans* genome (WBcel235) via HISAT2 v2.2.1, and quantified with StringTie v2.2.1. Differential expression analysis was performed using DESeq2 v1.38.3 (R Bioconductor) with |log_2_FC| ≥1 and an adjusted *p*-value < 0.05 indicated the significance thresholds.

### 2.9. Statistical Analysis

Data are presented as means ± SD. Statistical analyses were conducted using Statistical Product and Service Solutions (SPSS) 26.0 (IBM, Armonk, NY, USA) for *t*-tests and ANOVA, GraphPad Prism 9.0 (GraphPad Software, San Diego, CA, USA) for survival curve analysis (Logrank Mantel–Cox test), and R 4.3.1 (R Foundation) for transcriptomic data processing. Significance levels are indicated in the figures (* *p* < 0.05, ** *p* < 0.01).

## 3. Results

### 3.1. Ni Exposure Impaired the Development, Reproductive Capacity, and Food Preference of Caenorhabditis elegans

**Figure 1:**
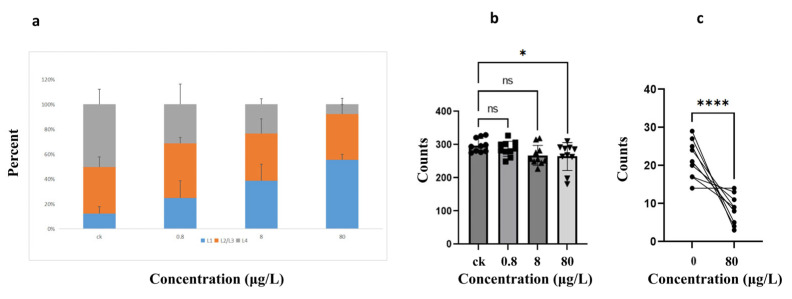
Tests of development, reproductive capacity, and food preference of *C. elegans* after exposure to Ni^2+^ at concentrations of 0.8, 8, and 80 μg/L, as well as the untreated control. (**a**) Percentage of nematodes at each developmental stage (L1, L2–L3, L4) after 72 h of Ni exposure. (**b**) Reproductive capacity assay of nematodes after 72 h of Ni exposure. The *Y*-axis represents the total number of eggs laid. (**c**) Acute (2 h) food preference assays. The *Y*-axis represents the number of nematodes in Regions A (OP50) and B (OP50 + 80 μg/L Ni^2+^), respectively. * *p* < 0.05, **** *p* < 0.000 in comparison with control. ns indicates no significant difference compared with the control group.

Synchronized *C. elegans* populations were exposed to control (OP50 only) or exposure groups (0.8, 8, or 80 μg/L of Ni^2+^) for 72 h to assess developmental toxicity. The larval stage distribution (L1, L2–L3, L4) was quantified as a percentage relative to the total population (L1 + L2–L3 + L4). As shown in Figure 1a, Ni^2+^ exposure disrupted the normal development of the nematode. Compared with the control group, the development of nematodes in the Ni-exposed groups were inhibited and showed a dose-dependent relationship. After exposure of the nematodes to 80 μg/L Ni, the proportion of L1 stage nematodes increased by 3.8 times compared to the control group. And the observed dose/response relationship was consistent with the inhibitory effect of Ni on early plant development. Germination of ground cover legumes (*Coronilla varia*) [43,44] and wheat (*Triticum aestivum* L. em thell.) cultivars was decreased by Ni^2+^ exposure [45]. Nickel (Ni^2+^) stress resulted in the inhibition of photosynthesis and reduction in seedling growth in *Aegilops tauschii* [46]. Our findings extend these toxicological patterns to metazoans, confirming that exposure to nickel ions can disrupt the critical developmental stages of different species.

To study Ni-induced reproductive toxicity, synchronized N2 populations were exposed to Ni^2+^ (0.8, 8, and 80 μg/L) in NGM plates. Following 72 h of exposure, 80 μg/L Ni^2+^ significantly reduced nematode egg production, suggesting that high concentrations of Ni are toxic to reproduction. Similarly, exposure to CdCl_2_ (30 μM) significantly reduced the number of offspring of *C. elegans* and prolonged its reproductive cycle [47].

Furthermore, this study evaluated the impact of Ni exposure on food preference in *C. elegans* to determine whether environmental Ni contamination alters food selection behaviors, thereby influencing habitat choice, population migration dynamics, and ultimately species population density and community biodiversity in Ni-polluted ecosystems. As shown in Figure 1c, nematodes exhibited avoidance behavior in response to Ni^2+^. According to studies by Catharine H. Rankin and G.E. Morrison et al., *C. elegans* utilizes olfactory cues to assess food quality and regulate foraging strategies—a behavior termed food odor-based decision-making [48,49]. This chemosensory mechanism allows nematodes to avoid toxic substrates or prioritize nutrient-rich environments [50,51]. Hence, our results indicate that the long-term presence of Ni may affect the development of the nematode population.

### 3.2. Ni Exposure Effects on Lifespan and Aging Processes of Caenorhabditis elegans

**Figure 2:**
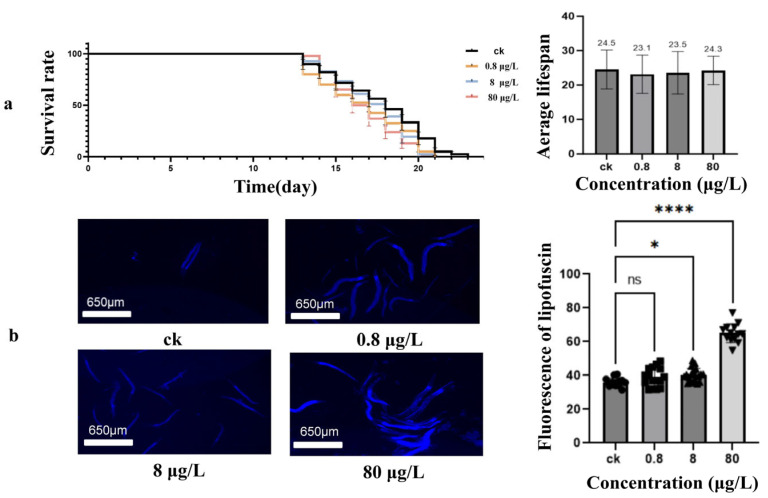
Effects of different concentrations of Ni on the lifespan and lipofuscin accumulation in *C. elegans.* (**a**) Survival curves (**left**) and the mean lifespan (**right**) of N2 worms treated with different concentrations of Ni. In each treatment condition, the number of worms was greater than 50. (**b**) Lipofuscin accumulation imaging (**left**) and the fluorescence intensity obtained through statistical analysis using ImageJ (**right**) of N2 worms treated with different concentrations of Ni^2+^. * *p* < 0.05, **** *p* < 0.000 in comparison to control. ns indicates no significant difference compared with the control group.

Compared with most other commonly used species, the lifespan test of *C. elegans* is relatively simple and can be easily analyzed [52]. Synchronized treatment populations of *C. elegans* were exposed to Ni^2+^ (0.8 μg/L, 8 μg/L, and 80 μg/L) to assess longevity effects. As shown in Figure 2a, the average survival time of the nematodes maintains consistency, from 24.5 ± 1.2 days in the control group (*n* = 51) to 23.1 ± 1.1 days (*n* = 56), 23.5 ± 1.0 days (*n* = 53), and 24.3 ± 1.3 days (*n* = 56) at 0.8, 8, and 80 μg/L group, respectively. The log-rank test—a standard statistical method for comparing survival distributions between groups—revealed no significant reduction in lifespan (χ^2^ = 5.8, *p* = 0.12). The Kaplan–Meier survival curve and average life calculations reveal that the Ni concentrations employed in this study were relatively low and did not directly induce significant lifespan impairment in *C. elegans*.

Since lipofuscin is commonly used in the study of aging, oxidative stress, and mechanisms of lifespan regulation [42], we studied the accumulation of lipofuscin in *C. elegans* after exposure to Ni. As shown in Figure 2b, exposure to Ni leads to an increase in the accumulation of lipofuscin in 3-day-old adult nematodes. Compared with the control group, the groups exposed to 8 and 80 μg/L of Ni^2+^ exhibited a remarkable increase in lipofuscin content (*p*  <  0.05). These findings suggest that although the current exposure dose of Ni (0.8–80 μg/L) does not affect nematode lifespan, it can accelerate the aging process.

### 3.3. Transcriptome Analysis After Ni Exposure

**Figure 3:**
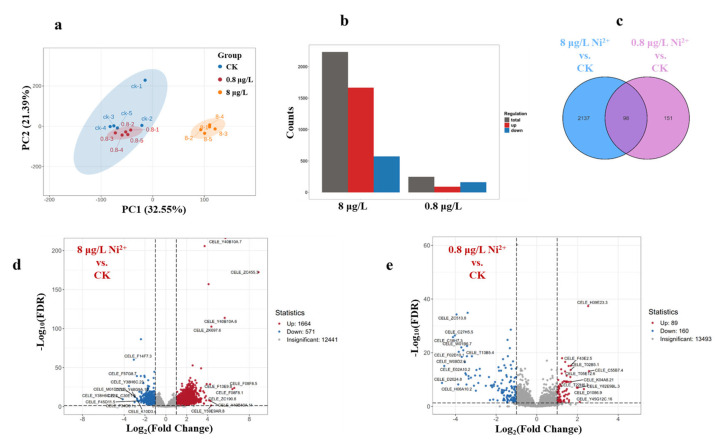
Differences in gene expression induced by different concentrations of Ni^2+^. (**a**) Principal component analysis (PCA). Multivariate separation of transcriptomes along PC1 (32.55% variance) and PC2 (21.38%), revealing distinct clustering of control (CK), 0.8 μg/L, and 8 μg/L groups. Ellipses mark 95% confidence intervals. (**b**) Histogram of the number of differentially expressed genes (DEGs) at different concentrations of Ni^2+^. Bar plot quantifying upregulated (red), downregulated (blue), and total DEGs across comparisons. (**c**) Venn diagram of all significant genes. (**d**) Volcano plot of 8 μg/L Ni^2+^ exposure group with all significant genes that are differentially expressed. (**e**) Volcano plot of 0.8 μg/L Ni^2+^ exposure group with all significant genes that are differentially expressed. In the volcano plot, red dots represent upregulated differentially expressed genes (DEGs), blue dots represent downregulated DEGs, while gray dots indicate genes that did not show significant expression changes.

To elucidate the mechanism underlying Ni^2+^ toxicity, we collected whole nematodes and conducted transcriptome sequencing (RNA-Seq) analysis. According to the above results and taking into account the environmental concentration, the Ni^2+^ exposure concentrations of 0.8 and 8 μg/L were selected. In the data analysis of RNA-Seq, principal component analysis (PCA) was one commonly used dimensionality reduction and visualization technique used to analyze the differences between groups. In Figure 3a, the PCA plot shows that PC1 and PC2 accounted for 32.55% and 21.38% of the variations, respectively. The samples were clearly divided into two groups along the PC1 axis, ck (control group) and the 8 μg/L of Ni^2+^ exposure group. Among them, the 0.8 μg/L of Ni^2+^ exposure group and the control group were clustered together in one large group, indicating that the 0.8 μg/L of Ni^2+^ exposure group had little effect on the transcriptome of the nematodes. As shown in Figure 3b–e, compared to untreated worms, the worms exposed to 0.8 μg/L of Ni^2+^ exhibited a total of 249 differentially expressed genes (DEGs), with 89 upregulated and 160 downregulated, while the worms exposed to 8 μg/L of Ni^2+^ exhibited more significant transcription disturbances, with a total of 2235 DEGs (1664 upregulated, 571 downregulated). Among them, the two exposure groups shared 98 DEGs (Figure 3c). The differential gene map showed that the upregulation of genes was dominant, which suggested that the experimental treatment activated specific pathways. The distinct transcriptional profiles observed between low-dose (0.8 μg/L) and high-dose (8 μg/L) treatments suggest a dose-dependent alteration in transcriptional regulation.

### 3.4. Gene Ontology (GO) Enrichment Reveals Ni^2+^ Treatment Impacts C. elegans Stress Response, Development, and Metabolism

**Figure 4:**
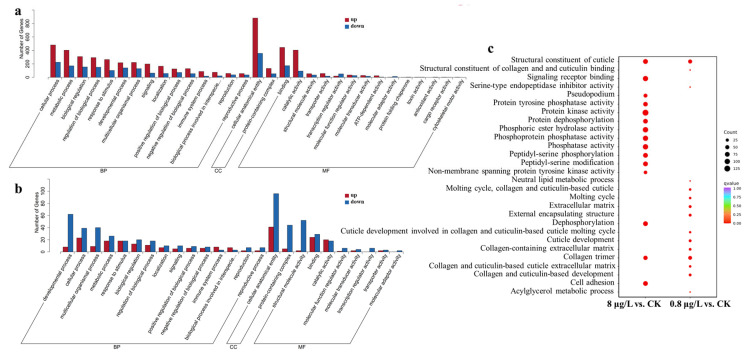
Gene Ontology (GO) pathway diagram of Ni treatment at different concentrations. (**a**) GO enrichment analysis of DEGs for 8 μg/L of Ni^2+^ exposure group. (**b**) GO enrichment analysis of DEGs for 0.8 μg/L of Ni^2+^ exposure group. In Figures a and b, only the top 15 GO terms for each of the three major categories—BP, CC, and MF (if there are fewer than 15 GO terms, all are displayed)—are shown. BP, biological process; CC, cellular component; MF, molecular function. (**c**) Multi-combination GO enrichment and scatter point diagram; only the top 10 GO terms (if there are fewer than 10 GO terms, all are displayed) are shown. The abscissa is the comparative combination; the ordinate is the enrichment pathway; the size of the point represents the number of differential genes enriched in the pathway; the larger the point, the more genes enriched in the pathway; the color of the point represents the significance value of enrichment for the pathway; the color of the point represents the significant value of enrichment for the pathway; and the redder the color of the point, the more significant the enrichment.

To further explore the mechanism of toxicity exhibited by N2 nematodes after exposure to different concentrations of Ni^2+^, gene enrichment analysis was conducted on the transcriptome data. Through Gene Ontology (GO, a framework for gene functional classification) enrichment analysis of DEGs, this study revealed significant effects of Ni^2+^ treatment on the key biological processes of *C. elegans*, mainly focusing on three categories, biological process (BP), molecular function (MF), and cellular component (CC). As shown in Figure 4a,b, the upregulated genes accounted for the majority among the GO enrichment pathways in the 8 μg/L of Ni^2+^ exposure group. However, the situation was exactly the opposite in the 0.8 μg/L of Ni^2+^ exposure group. After exposure to 8 μg/L of Ni^2+^, the DEGs of the nematodes were mainly enriched in the structural constituent of the cuticle, signaling receptor binding, protein tyrosine phosphatase activity, Pseudopodium, protein dephosphorylation, phosphoric ester hydrolase activity, phosphoprotein phosphatase activity, protein kinase activity, phosphatase activity, peptidyl-serine phosphorylation, peptidyl-serine modification, dephosphorylation, non-membrane spanning protein tyrosine kinase activity, collagen trimer, and cell adhesion GO pathways. Protein dephosphorylation enrichment may affect muscle contraction and signal transduction by regulating kinase/phosphatase homeostasis. After exposure to 0.8 μg/L of Ni^2+^, the DEGs in nematodes were primarily enriched in the following GO pathways: structural constituent of collagen and cuticulin binding, serine-type endopeptidase inhibitor activity, neutral lipid metabolic process, molting cycle, collagen and cuticulin-based cuticle, molting cycle, extracellular matrix, external encapsulating structure, cuticle development involved in collagen and cuticulin-based cuticle molting cycle, cuticle development, collagen-containing extracellular matrix, collagen and cuticulin-based cuticle extracellular matrix, collagen and cuticulin-based development, and acylglycerol metabolic process (Figure 4c). It has been found that a decrease in collagen on the top surface of the epidermis can cause selective aging of the photoreceptor neurons in *C. elegans*, leading to excessive dendritic branching and related functional defects [53].

### 3.5. Kyoto Encyclopedia of Genes and Genomes (KEGG) Enrichment Unveils Ni^2+^ Treatment Effects on C. elegans Metabolism, Signaling, and Aging Pathways

**Figure 5:**
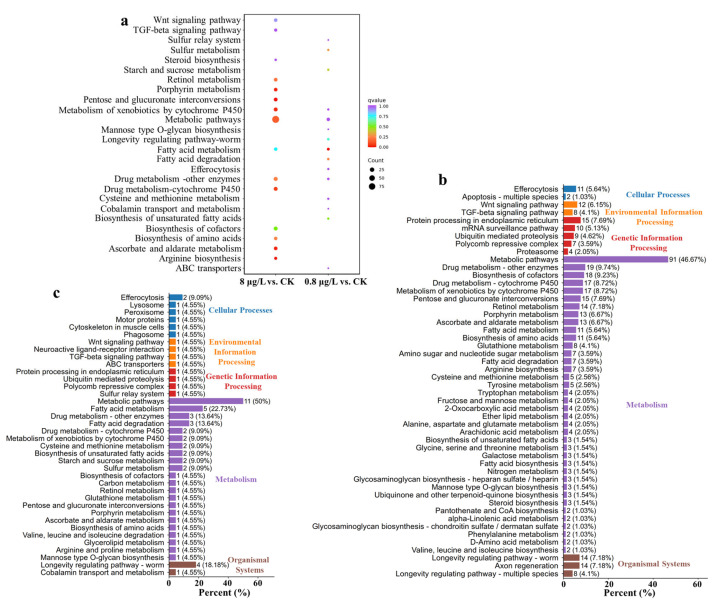
Kyoto Encyclopedia of Genes and Genomes (KEGG) pathway diagram of Ni treatment at different concentrations. (**a**) The top 15 significantly enriched KEGG pathways of DEGs with different Ni^2+^ treatment. For multi-combination KEGG enrichment and scatter plots, the abscissa is the comparative combination; the ordinate is the enrichment pathway; the size of the point represents the number of genes that are enriched in the pathway; the larger the point, the more genes are enriched to the pathway; the color of the point represents the significance value of the enrichment of the pathway; and the redder the color of the point, the more significant the enrichment. (**b**) KEGG enrichment histogram of 8 μg/L Ni^2+^ treatment group. (**c**) KEGG enrichment histogram of 0.8 μg/L Ni^2+^ treatment group. In figures (**b**,**c**), the top 50 significantly enriched KEGG pathways are shown (if there are fewer than 50 KEGG terms, all are displayed). The abscissa represents the number of differential genes annotated to the pathway, the ordinate represents the name of the KEGG pathway, the number in the figure represents the number of differential genes annotated to the pathway, the ratio of the number of differential genes annotated to the pathway to the total number of annotated differential genes is in parentheses; and the rightmost label represents the classification to which the KEGG pathway belongs.

Kyoto Encyclopedia of Genes and Genomes (KEGG, a pathway analysis database) enrichment analysis revealed the significant effects of Ni^2+^ treatment on the key metabolism and signaling pathways of *C. elegans*. DEGs are mainly enriched in metabolic regulation, signal transduction, stress response, and longevity-related mechanisms (Figure 5). After exposure to 8 μg/L of Ni^2+^, the top 15 KEGG pathways enriched by DEGs include the Wnt signaling pathway, TGF-beta signaling pathway, steroid biosynthesis, retinol metabolism, porphyrin metabolism, pentose and glucuronate interconversions, metabolism of xenobiotics by cytochrome P450, metabolic pathways, fatty-acid metabolism, drug metabolism—other enzymes, drug metabolism—cytochrome P450, biosynthesis of cofactors, biosynthesis of amino acids, ascorbate and aldarate metabolism, and arginine biosynthesis. In the 0.8 μg/L of Ni^2+^ exposure group, the top 15 KEGG pathways enriched by DEGs include sulfur relay system, sulfur metabolism, starch and sucrose metabolism, metabolism of xenobiotics by cytochrome P450, metabolic pathways, mannose type O-glycan biosynthesis, longevity-regulating pathway—worm, fatty-acid metabolism, fatty-acid degradation, efferocytosis, drug metabolism—other enzymes, cysteine and methionine metabolism, cobalamin transport and metabolism, biosynthesis of unsaturated fatty acids, and ABC transporters. Compared to the control group, the types of pathways enriched for 8 and 0.8 μg/L of Ni^2+^ were similar, but the numbers of pathways enriched and DEGs were different (Figure 5b,c). There were 22 pathways in the metabolism of the 0.8 μg/L of Ni^2+^ exposure group but 38 pathways in the metabolism of the 8 μg/L of Ni^2+^ exposure group.

## 4. Discussion

This study investigated the toxic effects of Ni^2+^ exposure on *C. elegans*. The obtained experimental results demonstrated that exposure to Ni^2+^ at environmentally relevant concentrations impaired the physiological functions of the nematodes, including growth, reproductive capacity, food preference, and lipofuscin accumulation (Figure 1 and Figure 2). Transcriptomic analysis further identified DEGs and key metabolic pathways associated with Ni^2+^ toxicity. Ni exposure triggered a hierarchical toxicity cascade in *C. elegans* in a concentration-dependent manner. High-dose exposure (8 μg/L of Ni^2+^) provokes widespread transcriptional dysregulation (2235 DEGs); for example, *ugt-3*, *numr-1*, *numr-2*, *comt-5*, *comt-4*, *gst-35*, *gst-21*, and *gst-9* were significantly upregulated (Figure 3). Gao et al. [54] found that *gst-35* overexpression significantly reduced lifespan, impaired development and growth, and substantially diminished reproductive capacity, physical fitness, and stress resistance. *C. elegans numr-1/2* (nuclear-localized metal-responsive genes) constitute an identical gene pair encoding a nuclear protein. This protein has been previously shown to be activated by Cd (100 μM) and disrupted by the integrator RNA metabolism complex [55,56]. Although no significant changes were observed in the worm phenotype at the 0.8 μg/L Ni exposure concentration, a total of 249 DEGs were found by RNA-Seq. *Col* genes (e.g., *col-90*, *col-41*, *col-17*, *col-50*, *col-85*, *col-72*, *col-45*, *col-84*, *col-164*, *col-44*, *col-158*, *col-35*, *col-51*, *col-37*, *col-40*, *col-123*, *col-2*, *col-183*, *col-185*, *col-43*, *col-36, col-102*, *col-114, col-149, col-128*) and *grl* genes (*grl-17*, *grl-9*, *grl-20*, *grl-25*, *grl-23*) were significantly downregulated, which suggested that cuticle collagen synthesis was disrupted by Ni. Similar results were observed after worms were exposed to copper [57].

GO enrichment analysis showed Ni exposure greatly influenced BP and MF (Figure 4) and that both concentrations of Ni exposure affect growth and development. After exposure to Ni^2+^, the metabolism of xenobiotics by cytochrome P450 and drug metabolism pathways were enriched, suggesting that Ni exposure may activate detoxification mechanisms typically involved in neutralizing exogenous toxicants (Figure 5). Fatty acid-related metabolism and amino acid-related biosynthesis process indicated Ni may affect energy metabolism and feeding behavior and indirectly lead to growth and development delay. The dysregulation of the signal transduction-related pathways and the Wnt and TGF-β signaling pathways is the core mechanism affecting the development and reproduction of nematodes [58]. After exposure to 8 μg/L of Ni^2+^, the expression of *cwn-2*, *cfz-2*, *skr-9*, *skr-13*, and *Y47D7A.15* was downregulated in nematodes, while the expression of *gskl-2*, *gskl-1*, and *F21F3.2* was upregulated. These alterations in gene expression may disrupt cell migration and lead to developmental abnormalities [59]. The expression of receptor gene *R04A9.7*, *skr-10*, *skr-13, skr-9*, *skr-14*, *skr-8*, *skr-7*, and *skr-21* was downregulated, which indicated that Ni exposure disrupted the meiosis, cell proliferation, and morphogenesis capabilities of the nematodes [60]. In the longevity-regulating pathway (worm), *ins-7* was upregulated; R04A9.7 was downregulated; downstream interference was found with hsp-12.6, hsp-16.2, and hsp-16.41; oxidative damage was reduced, and life was prolonged [61]. Abnormalities in pathways related to cellular structure and function, extracellular matrix, and membrane structure were closely related to the phenotypic decline of the nematodes. The expression of collagen genes was downregulated after exposure to 8 μg/L Ni^2+^, weakening the structural integrity of the epidermis and reproductive tract, resulting in reduced oviposition efficiency [62]. Dual perturbations from upregulated Wnt ligands and downregulated TGF-β receptors likely disrupted cell-cycle checkpoints [63], altering developmental timing and cell fate determination, while widespread downregulation of collagen-encoding genes directly compromised cuticle and reproductive tract structural integrity. The expression levels of genes related to lysosomes (*lipl-3*, *lipl-4*, *lipl-7*, and *lbp-7*) were abnormal in Ni-treated worms, resulting in intestinal lipofuscin accumulation—a hallmark of aging [64].

Although this study provides information on the phenotypic changes and transcriptomic alterations in *C. elegans* after exposure to environmental concentrations of Ni, there are still some limitations. For instance, Ni exposure concentrations (0.8–80 μg/L) in experimental settings cover some environmentally relevant concentrations but do not cover higher doses or lower doses, which may limit the full interpretation of Ni toxicity thresholds and nonlinear dose effects. Transcriptomic analysis reveals changes in key genes and pathways, but there is a lack of integrated validation of proteomics or metabolomics to clarify whether changes in transcriptional levels directly lead to dynamic changes in functional protein expression or metabolites. Experiments are performed under standard laboratory conditions and do not simulate the combined exposure effects of Ni on other contaminants in the real environment or the regulatory effects of environmental factors on Ni bioavailability, which may lead to biased toxicity assessments. Future research needs to combine multi-omics technology, environmental simulation experiments, and gene editing methods to further reveal the spatiotemporal dynamic mechanism of Ni toxicity and its ecological health risks.

## 5. Conclusions

This study comprehensively elucidates the multi-dimensional toxicity of Ni in *C. elegans*. Experimental results demonstrated that exposure to Ni^2+^ at environmentally relevant concentrations impaired the physiological functions of the nematodes, including growth, reproductive capacity, food preference, and lipofuscin accumulation. Transcriptomic profiling identified key genes and pathways linking collagen metabolism defects, energy supply disruption, and lysosomal dysfunction to phenotypic outcomes. These findings underscore Ni’s ecological risks at environmentally relevant concentrations and highlight conserved molecular targets for predictive toxicology. Future research needs to combine multi-omics technology, environmental simulation experiments, and gene editing methods to identify Ni’s specific toxicity mechanisms and further explore its ecological health risks.

## Data Availability

The data presented in this study are available on request from the corresponding author.

## Supplementary Materials

The following supporting information can be downloaded at: https://www.mdpi.com/article/10.3390/toxics13110930/s1, Supplementary File S1: Supplemental Methods: Transcriptomic Analysis.
