# Supplementary material for: Transcriptomics-Based Toxicological Study of Nickel on Caenorhabditis elegans
Source: Toxics. 2025 Oct 30;13(11):930. doi: 10.3390/toxics13110930 (PMC12656052; doi:10.3390/toxics13110930)
Supplement: Supplementary file 1 [file toxics-13-00930-s001.zip › toxics-3552647-supplementary.pdf]

Supplementary Materials

Transcriptomics-Based Toxicological Study of Nickel on *Caenorhabditis elegans*

Yutao He[1], Yunfei Long[1], Jingwen Wang[1], Qinfen Li[2], Beibei Liu[2], Dandan Li[1,*] and Shunqing Xu[1,*]

Supplemental Methods: Transcriptomic Analysis

Transcriptomes of *C. elegans* were prepared as previously described[1]. Specifically, synchronized *C. elegans* were cultured on NGM plates with different Ni^{2+} treatments until reaching Day 3. Then, the nematodes were collected and washed by centrifugation (Multifuge X1R Pro, Thermo Scientific) and quickly frozen in liquid nitrogen for transcriptome sequencing analysis. Total RNA was extracted from Ni^{2+}-exposed nematodes using the Trizol method. After successful extraction, RNA was dissolved by adding 50 μL of diethyl pyrocarbonate (DEPC)-treated water. Then, RNA quality and quantity were assessed using a Qubit fluorometer (Qubit 4.0, Thermo Fisher Scientific, Waltham, USA) and Qsep400 Bio-Fragment Analyzer (BiOptic Inc., Taiwan, China). RNA integrity was confirmed with RNA Integrity Number (RIN) ≥8.0.

Based on the structural feature that most eukaryotic mRNAs have a poly(A) tail, mRNA purification and fragmentation were performed on the total RNA extracted in the previous step using Hieff NGS® Ultima Dual-mode RNA Library Prep Kit (Premixed Version) from Yeasen Biotechnology (Shanghai) Co., Ltd. according to the product manual. Specific operations can be referred to in Yeasen Biotechnology's official technical documents. The fragmented mRNA was subjected to first-strand cDNA synthesis using random hexamer primers (short single-stranded DNA oligonucleotides of random sequence, each six nucleotides in length). This was followed by second-strand synthesis using a strand-specific protocol (which preserves the directional information of transcripts) that incorporates dUTP to label the second strand. The resulting double-stranded cDNA underwent end repair, dA-tailing, and adapter ligation; was size-selected for inserts of 250-350 bp; and was then PCR-amplified and purified to construct the initial library. Subsequently, the quality-controlled double-stranded library was converted into a single-stranded circular DNA library. The single-strand circularization technology of Beijing Genomics Institution (BGI, China) converts double-stranded DNA libraries into highly stable single-stranded circular DNA libraries through core steps such as high-temperature denaturation, asymmetric primer design, T4 ligase-catalyzed circularization, and phi29 polymerase-based rolling circle amplification. Specific operations can be referred to in BGI Genomics' official technical documents. The final library was amplified using phi29 polymerase to generate DNA nanoballs (DNBs), each containing over 300 copies per molecule. The DNBs were then loaded into a sequencing chip and processed on the BGI sequencing platform. All steps were conducted under RNase-free conditions with strict adherence to manufacturer protocols and quality control measures.

Raw sequencing data were processed using FastQC v0.12.1 for quality control, aligned to the *C. elegans* genome (WBcel235) *via* HISAT2 v2.2.1, and quantified with StringTie v2.2.1. Differential expression analysis was performed using DESeq2 v1.38.3 (R Bioconductor) with $|\log_2 FC| \geq 1$ and adjusted p-value <0.05 as significance thresholds.

References

[1] Ren, H.S.; Yin, K.; Lu, X.H.; Liu, J.J.; Li, D.D.; Liu, Z.J.; Zhou, H.L.; Xu, S.Q.; Li, H.Z.; Synergy between nanoplastics and benzo*a*pyrene promotes senescence by aggravating ferroptosis and impairing mitochondria integrity in *Caenorhabditis elegans*, *Sci. Total Environ.*, **2024**, 946.
